# Physical Activity Level Following Resistance Training in Community-Dwelling Older Adults Receiving Home Care: Results from a Cluster-Randomized Controlled Trial

**DOI:** 10.3390/ijerph18136682

**Published:** 2021-06-22

**Authors:** Hilde Bremseth Bårdstu, Vidar Andersen, Marius Steiro Fimland, Lene Aasdahl, Hilde Lohne-Seiler, Atle Hole Saeterbakken

**Affiliations:** 1 Department of Sport, Food and Natural Sciences, Faculty of Education, Arts and Sports, Western Norway University of Applied Sciences, 6851 Sogndal, Norway; vidar.andersen@hvl.no (V.A.); atle.saeterbakken@hvl.no (A.H.S.); 2 Department of Neuromedicine and Movement Science, Faculty of Medicine and Health Sciences, Norwegian University of Science and Technology, 7030 Trondheim, Norway; marius.fimland@ntnu.no; 3 Unicare Helsefort Rehabilitation Centre, 7112 Hasselvika, Norway; lene.aasdahl@ntnu.no; 4 Department of Public Health and Nursing, Faculty of Medicine and Health Sciences, Norwegian University of Science and Technology, 7030 Trondheim, Norway; 5 Department of Sport Science and Physical Education, Faculty of Health and Sport Science, University of Agder, 4604 Kristiansand, Norway; hilde.l.seiler@uia.no

**Keywords:** strength training, elderly, independent living, exercise, physical behavior

## Abstract

Older adults’ physical activity (PA) is low. We examined whether eight months of resistance training increased PA level in community-dwelling older adults receiving home care. A two-armed cluster-randomized trial using parallel groups was conducted. The included participants were >70 years and received home care. The resistance training group performed resistance training using body weight, elastic bands, and water canes twice per week for eight months. The control group was informed about the national PA guidelines and received motivational talks. The ActiGraph GT3X+ accelerometer was used to estimate PA. Outcomes included total PA (counts per minute), sedentary behavior (min/day), light PA (min/day), moderate-to-vigorous PA (min/day), and steps (mean/day). Between-group differences were analyzed using multilevel linear mixed models. Twelve clusters were randomized to either resistance training (7 clusters, 60 participants) or the control group (5 clusters, 44 participants). A total of 101 participants (median age 86.0 (interquartile range 80–90) years) had valid accelerometer data and were included in the analysis. There were no statistically significant between-group differences for any of the PA outcomes after four or eight months. This study offers no evidence of increased PA level following resistance training in older adults with home care.

## 1. Introduction

Physical Activity (PA) is important for successful ageing [1], reducing the risk of several non-communicable diseases [2,3] and all-cause mortality [4]. In Norway, only three out of ten community-dwelling older adults above 65 years achieved the recommended 150 min/week of moderate-to-vigorous-PA (MVPA) [5], decreasing to 5.6% in individuals above 80 years [6]. Low PA levels are related to reduced physical function and independence [3,7]; thus, maintaining and/or increasing PA levels into old age is important.

With increasing age, muscle strength, physical function (e.g., ability to rise from a chair and walking), and PA levels gradually decline [3]. Many older adults experience this as a vicious cycle; poor muscle strength is related to impaired physical function [3,8] and low PA levels [9], and impaired physical function is related to low PA [7,10]. For example, there is an association between muscle strength and walking speed [8], and walking is found to contribute greatly to older adults’ daily PA [11,12]. While the benefits of resistance training on muscle strength and physical function in older adults are well established [3,13], the impact on PA levels is unclear [14,15,16,17,18]. Studies reporting no change in PA levels following resistance training [14,16,17,18] have failed to improve muscle strength and/or physical function. In contrast, in one study including institutionalized older adults where both leg strength and physical function improved, PA levels were found to increase [15]. Whether resistance training programs that improve muscle strength and physical function lead to a concurrent increase in PA levels should be more thoroughly investigated. Furthermore, time spent in sedentary behavior (SB) and light PA (LPA) have received much attention over the last decade due to their association with physical health and well-being in older adults [19,20]. Despite this, we are only aware of one study that has investigated whether engaging in resistance training is effective for reducing time spent in SB and increasing LPA, and the intervention was for a short duration (10 weeks) and the sample small [16].

The World Health Organization emphasizes the need for tailored resistance training programs that can be performed in older adults’ key-settings, such as at home or in care centers [21]. Resistance training programs utilizing low-cost equipment (e.g., body weight, elastic bands) hold great potential, even for the oldest old (>80 years) [16,18]. These programs might overcome older adults’ barriers, such as affordability and lack of availability [22], as training can be performed everywhere. Moreover, they facilitate the inclusion of functional movements and exercises that replicate older adults’ daily activity patterns. Therefore, such resistance training programs, which aim to strengthen weakened muscles and improve physical function, could potentially increase PA, making movement easier.

We recently demonstrated that eight months of resistance training improved leg muscle strength and physical function in community-dwelling older adults receiving home care [23]. If the PA level increases as a natural consequence of improved leg strength and physical function, i.e., by making movement easier, resistance training can offer a wider range of benefits than previously established. The present study reports secondary outcomes on whether PA levels increased in older adults performing resistance training as compared to a control group receiving PA counselling. It was hypothesized that PA levels would increase more in the resistance training group compared to the control group.

## 2. Materials and Methods

### 2.1. Trial Design

The present paper used data from the Independent Self-Reliant Active Elderly (ISRAE) study, a two-armed, open-label, parallel group cluster-randomized controlled trial (RCT) performed in three Norwegian municipalities (Sogndal, Luster, and Leikanger) from August 2016 to June 2019. The intervention was for a period of eight months, and compared a resistance training group (RTG) to a control group (CG) receiving PA counselling. After the end of the intervention, we followed the participants for two years. The primary outcome of ISRAE is the ability to live independently and be self-reliant at home at the end of the two-year follow-up. We have previously reported the intervention effect on the secondary outcomes physical function and muscle strength [23]; thus, the method sections will partly overlap. This paper reports the effect of the intervention on PA levels four and eight months after study inclusion (secondary outcomes).

The study was evaluated by the Regional Committee for Medical and Health Research Ethics South East and the Norwegian Centre for Research Data (Bergen, Norway) (2016/51 and 49361/s/AGH, respectively) and was conducted in accordance with the Declaration of Helsinki and Norwegian laws and regulations. The study was registered in the ISRCTN registry (1067873, retrospectively registered). The results are presented according to the CONSORT statement extension to cluster-randomized trials [24]. Participants received written and oral information about the trial before signing a written informed consent form.

### 2.2. Participants

Participants were identified through the health care services in the three municipalities. Inclusion criteria were (i) age > 70 years, (ii) community-dwelling, and (iii) receiving home care due to medical and/or functional disabilities. Exclusion criteria were serious cognitive impairments (e.g., dementia, Alzheimer’s disease), diagnoses/conditions affecting testing or training, or contraindications for training from a medical doctor. In addition, we included seven participants below 70 years (median age 67 (range 63–69) years), who otherwise met the inclusion and exclusion criteria. This was to increase the sample size, and a revision was made to the initial eligibility criteria.

### 2.3. Intevention

The RTG performed two exercise sessions a week during the eight months intervention, which lasted from the end of September 2006 to the end of May 2017. Details about the resistance training program have been published previously [23]. Briefly, training sessions were supervised by trained exercise instructors and lasted for 30–45 min. The resistance training program utilized elastic bands (ROPES a/s, Aasgardstrand, Norway), body weight, and water canes, which are considered low-cost and easily available equipment. Exercises included rowing, squats, chest press, knee extension, biceps curl, shoulder press, and up-and-go (i.e., participant standing up from a chair, walking 3 m and turning around a cone, walking back and sitting back down). The first five weeks after baseline testing served as an introductory phase focusing on correct execution of the exercises at a submaximal intensity. Thereafter, we increased the volume and intensity progressively according to recommendations [25], and exercises were to be performed to fatigue (i.e., unable to perform additional repetitions with the correct technique). The intensity was individually tailored with chairs and water canes and by changing the elastic bands’ tension and thickness. The concentric phase was performed with high intentional velocity, while the eccentric phase was performed with slow, controlled intensity. Furthermore, the participants were urged to maintain their normal, habitual activity levels. Attendance was defined as the percentage of sessions met out of the total number of sessions.

Participants in CG received PA counselling in accordance with the national guidelines [26] and also received an educational brochure from the Ministry of Health and Care Services. Furthermore, every sixth week, they were visited or contacted by one of the researchers or research assistants. These conversations served to remind participants about the national PA guidelines and to motivate them to stay active.

### 2.4. Physical Activity Outcomes

The ActiGraph GT3X+ triaxial accelerometer (ActiGraph, LLC, Pensacola, FL, USA) was used to assess PA level, at participant level and at baseline after four and eight months. Participants were asked to wear the accelerometer in a belt over the right hip for at least 14 consecutive days and only to remove it when in contact with water or while sleeping. The accelerometer was initialized, and data were downloaded using ActiLife v.6.11 (ActiGraph, LLC, Pensacola, FL, USA). The sampling rate was 30 Hz, and data were analyzed in 10 s epochs. Vector Magnitude (VM) was used for analyses, and the ActiGraph low frequency extension (LFE) filter was applied. The LFE filter sets a lower frequency threshold for detecting accelerations, capturing slower movements often seen in older adults [27]. Non-wear time was defined as at least 90 consecutive min of zero counts, allowing for a 2 min interval of non-zero counts if accompanied by 30 min of consecutive zero up- or downstream [28]. The first day of wearing the accelerometer was excluded due to the risk of reactivity [29]. Files with at least 10 h of data for at least 4 days were considered valid [30]. Data between midnight and 6:00 a.m. were excluded.

Outcomes were total PA (TPA, counts per minute (cpm)), SB (min/day), LPA (min/day), MVPA (min/day), and steps (steps/day). The intensity thresholds were 0–199 cpm for SB [31], 200–1923 cpm for LPA [31,32], and ≥1924 cpm for MVPA [32] as suggested for older adults using VM. The number of steps was registered using the embedded pedometer function in GT3X+.

### 2.5. Randomization and Blinding

Participants were randomly allocated based on their geographical residency, i.e., participants living nearby belonged to the same cluster. Cluster randomization was chosen as it minimizes the risk of contamination and increases adherence. Twelve clusters (5–16 participants) were identified and allocated to RTG or CG. Clusters were randomized using a ratio of 3:2. The project leader conducted the randomization using the following procedure: (i) a number (1–16) was assigned to each cluster and clusters with ≥10 participants were weighted with two numbers; (ii) 60% of the participants (i.e., seven clusters) were allocated to RTG using a random numbers table; and (iii) the remaining participants (i.e., five clusters) were allocated to CG.

Due to practical concerns, we did not blind the researchers or research assistants. Furthermore, the nature of the intervention made it impossible to blind the participants and exercise instructors.

### 2.6. Statistical Analysis

The intention-to-treat principle was used to analyze the outcomes. Between-group effects were evaluated using multilevel linear mixed models. The baseline level of the outcome was generated by combining the baseline levels of the two groups [33]. Participant-id and cluster were entered as random effects, taking the dependency of repeated measures and cluster randomization into account. We included an interaction for group and time (baseline, four, and eight months). Normality was evaluated by visually inspecting the residuals of the outcomes. The outcomes for the groups at baseline after four and eight months were predicted using the estimates from the analyses.

Per-protocol analyses, including participants in RTG with more than 60% attendance, were performed. In addition, we performed some sensitivity analyses. First, we excluded participants under the age of 70 years, based on the initial eligibility criteria. Second, the combined baseline was removed, and we adjusted for the baseline value of the outcome (adjusted model 1). Lastly, as the main analyses were not adjusted for covariates, we conducted sensitivity analyses adjusting for accelerometer wear time, age, and BMI (adjusted model 2) to assess the robustness of our results.

Descriptive data are presented as mean and standard deviation (SD) or median and 25–75 percentiles (interquartile range, IQR). Results from the analyses are presented as estimated means and at 95% confidence intervals (CI). We calculated the intra-cluster correlation coefficients (ICC) as the between-cluster variation divided by total variation [34]. Statistical significance was set to a *p*-value of <0.05. STATA 15 (StataCorp. 2017. Stata Statistical Software: Release 15. College Station, TX, USA: StataCorp LLC) was used for analyses.

## 3. Results

**Figure 1:**
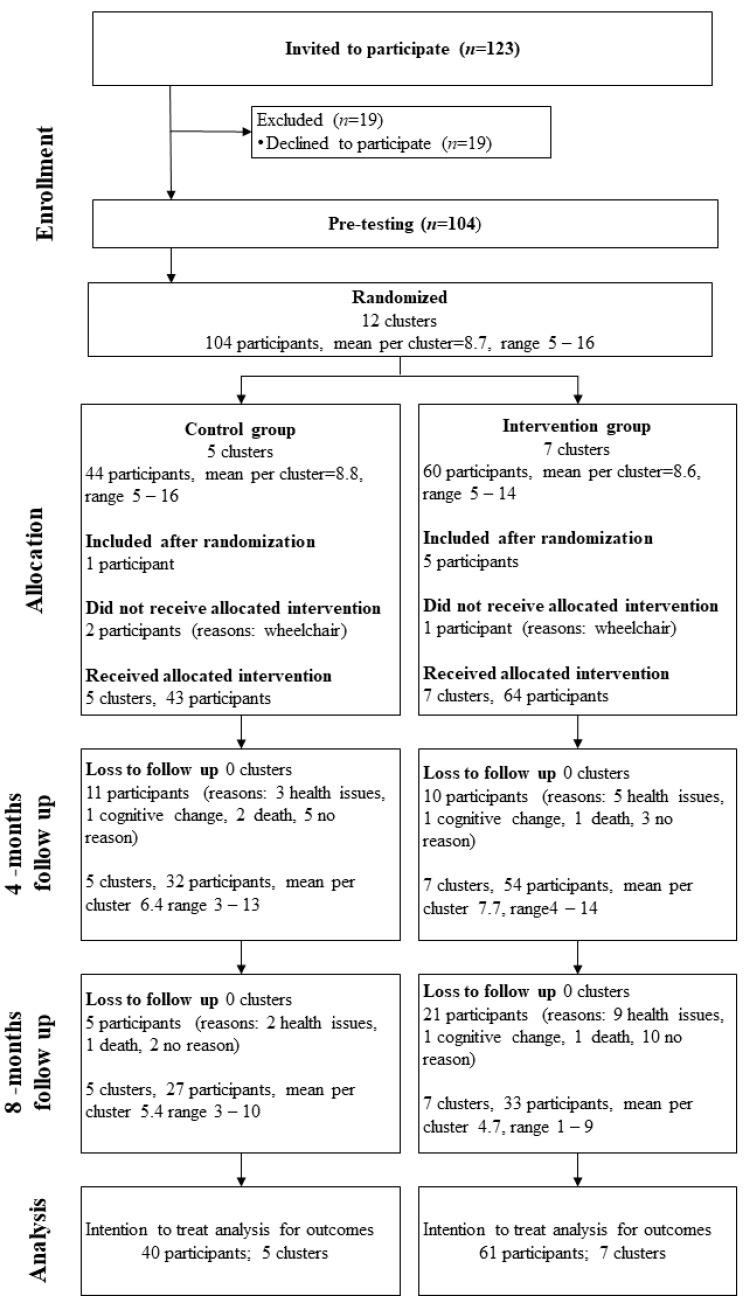
CONSORT (Consolidated Standards of Reporting Trails) flow chart of participant recruitment.

In total, 123 older adults meeting the inclusion criteria were invited to the study. Of those, 104 met for baseline testing and were split into 12 clusters. Furthermore, we included six participants after randomization. These participants were allocated to the correct cluster based on their geographical residency. Three participants in wheelchairs who could not carry out testing were excluded from analyses. Figure 1 illustrates the flow of participants throughout the study. We did not experience any adverse events.

### 3.1. Participant Characteristics

Table 1 presents baseline characteristics. The median age was 86 (IQR 80–90) years, and 60% were women. Assistive walking devices were used by 60%. Average attendance to resistance training was 51%, and 44% dropped out (RTG *n* = 31, CG *n* = 16). Six participants had no valid data at any measurement point and were not included in analyses. Participants without valid data were older (median 90 (IQR 87–90) years), and a higher number (67%) were male compared to those included in the analyses (median age 86 (IQR 80–90) years and 39% male). This left 101 participants divided into 12 clusters to be included in the analyses.

At baseline, mean accelerometer wear time was 809 (SD 74) min/day, and the median number of valid days was 13 (IQR 11–14) (Table 1).

### 3.2. Physical Activity Level

**Figure 2:**
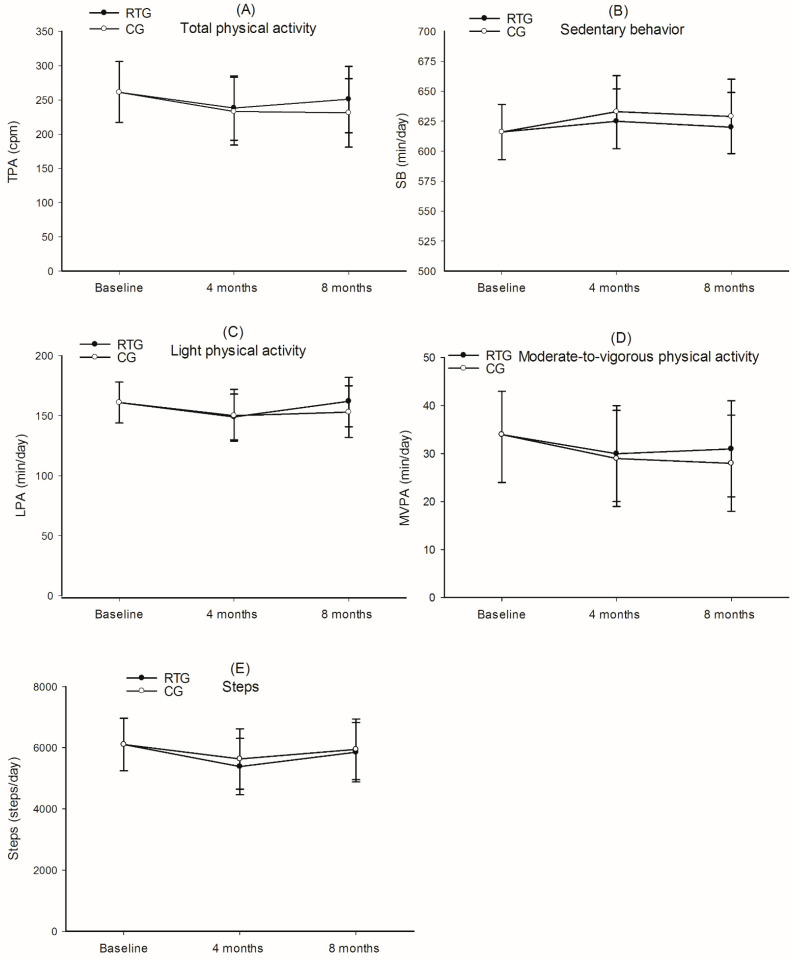
Changes in physical activity from baseline to four and eight months. Values are estimated means and 95% confidence intervals. RTG, resistance training group; CG, control group; Total PA, total physical activity; cpm, counts per minute; SB, sedentary behavior; LPA, light physical activity; MVPA, moderate-to-vigorous physical activity. (**A**) total physical activity, (TPA, cpm); (**B**) Sedentary behavior (SB, min/day); (**C**) Light physical activity (LPA, min/day); (**D**) Moderate-to-vigorous physical activity (MVPA, min/day); (**E**) Steps (steps/day).

There were no significant between-group differences for any of the PA outcomes from baseline to four and eight months (*p* = 0.371–0.880) (Figure 2A–E). Estimated mean TPA at baseline was 261 cpm (95% CI 217–306). Mean difference between RTG and CG was 5 cpm (95% CI−28–37) after four months and 20 cpm (95% CI−16–37) after eight months (Figure 2A). Estimated mean SB at baseline was 616 min/day (95% CI 593–639), and the mean difference between groups was 8 min/day (95% CI−38–22) after four months and 9 min/day (95% CI−42–23) after eight months, with CG spending slightly more time in SB compared to RTG (Figure 2B). For LPA, the estimated mean at baseline was 161 min/day (95% CI 144–178), and the mean difference between groups was−1 min/day (95% CI−21–18) in favor of CG after four months, and 8 min/day (95% CI−13–29) in favor of RTG after eight months (Figure 2C). For MVPA, the estimated mean at baseline was 34 min/day (95% CI 24–43), and the mean difference between groups was 1 min/day (95% CI−5–7) and 3 min/day (95% CI−3–10) after four and eight months, respectively (Figure 2D). The estimated mean steps per day were 6105 (95% CI 5247–6964). After four and eight months, the mean difference between the groups was−250 steps/day (95% CI−987–485) at four months and−90 steps/day (95% CI−896–716) at eight months, both in favor of CG (Figure 2E).

### 3.3. Per Protocol Analyses

There were no changes in the conclusions following the per-protocol analyses including participants in RTG with more than 60% attendance (Supplementary Table S1). Similarly, no changes in the conclusions were found following the other sensitivity analyses (Supplementary Tables S2–S4).

## 4. Discussion

There were no differences in TPA, SB, LPA, MVPA, or steps between participants in RTG receiving resistance training and participants in CG receiving PA counselling after four or eight months.

Our findings support a meta-analysis reporting that exercise interventions alone are not enough to stimulate older adults to increase their PA levels [35]. In a previous RCT, Oesen and co-workers [18] showed that elastic band resistance training had no effect on the number of steps taken by older adults living in a retirement facility (>80 years). Likewise, in a previous study from our lab [16], no change in PA levels was found in older adults receiving home care (>80 years) following 10 weeks of resistance training. However, these studies did not report improved leg strength [16,18] or physical function [16], which we expect is necessary to increase PA levels. In contrast, Fiatarone and colleagues [15] reported an effect of resistance training on PA levels, as well as leg strength and physical function, when compared to various leisure activities in institutionalized older adults (mean age 87.1 years). In the same study, leg strength and physical function improved. The contrasting findings may relate to differences in study design, training protocol (e.g., volume, frequency, equipment), populations included (e.g., different age and health status), and assessment of PA.

Over the last decade, the importance of SB and LPA for older adults’ health has been highlighted. Higher LPA and less time spent in SB are associated with several important health outcomes [19,20] and better physical function [10] in older adults. Consequently, the World Health Organization 2020 guidelines on PA and SB recommend both decreasing SB and increasing PA [36]. We found no effect of the resistance training program on SB or LPA. To our knowledge, only one previous study has included SB and LPA, and this study reported similar results [16].

Several factors could explain why we could not confirm our hypothesis. Although we designed the resistance training program according to recommendations [25], the attendance at the exercise sessions was low, with a mean of one session per week (51% attendance). Studies show that training programs of higher volume and frequency are more effective for older adults [37,38], which could explain our findings. Fiatarone and co-workers [15] reported large strength gains (26–216%) in institutionalized older adults, and our 16–18% increase in strength [23] might not have been sufficient to increase PA. Another possible explanation is that the resistance training program was not specific enough and that including strength exercises with a functional movement pattern do not necessarily transfer into increased PA. Although our aim was to investigate whether resistance training alone could be effective in increasing PA, combining resistance training with specific elements targeting PA, such as walking [39], balance training, and/or behavioral strategies (e.g., goal setting and education) [40,41] could be more effective. For example, walking interventions have been successful in increasing steps and muscle strength in older adults with osteoarthritis [39] and behavioral strategies [40] have been proposed as essential for changing PA). Furthermore, it has been suggested that older adults may reduce their spontaneous PA during the day to save energy for an upcoming training session or substitute spontaneous PA with inactivity due to muscle soreness and fatigue [42]. Lastly, participants’ characteristics (e.g., old age, home care, use of walking aids) could explain the lack of training effect considering that increasing PA level is less likely in those who are physically limited compared to healthy older adults [40].

Although using accelerometers to estimate PA are recommended, the lack of consensus on analytical approaches limits the comparability between studies [43]. Estimated MVPA was higher in our sample compared to previous estimates for older adults [5,6,44]. We used age-relative intensity thresholds instead of absolute thresholds to estimate MVPA. Relative thresholds are found to estimate more time in MVPA [45,46] and increase step count [46], which could explain our high PA levels. Furthermore, we estimated PA over 14 days, while the previously mentioned study reporting resistance training to be quite effective used 72 h [15]. Whether 72 h is adequate to capture an actual change in behavior, and not a spontaneous change, can be questioned [47].

Most of the PA estimates were at their lowest after four months. This was not surprising as the four months assessment was conducted in January and February, and the Norwegian winter is cold and snowy. Thus, this is most likely explained by seasonal variation in weather conditions [48].

The 14 days of objective PA measurement and age-appropriate intensity thresholds strengthens this study. Furthermore, we included estimates of SB and LPA, and showing the entire intensity spectrum of PA strengthens our study. Notwithstanding, some limitations should be acknowledged. First, dropout was high (44%), but this was not surprising due to the participants’ health and high age. We used multilevel mixed models which handles missing data by using all available data [33]. However, this relies on the assumption of missing at random and bias might be present as a result of the loss to follow up. We cannot rule out that some of the participants that dropped out without reason withdrew for reasons related to the training. Second, we included six participants after randomization, which could bias group allocation. Third, we did not estimate energy expenditure, making it impossible to conclude whether RTG used less of their maximal capacity during exercising and daily life activities (improved their stamina) after the intervention. Lastly, the sample size is small for a cluster RCT, and the study was not blinded. Thus, due to study limitations, these findings should be interpreted with some caution.

## 5. Conclusions

In conclusion, eight months of resistance training compared to PA counselling did not change TPA, SB, LPA, MVPA, or steps per day in community-dwelling older adults receiving home care. This study indicates that a resistance training program, utilizing low-cost, easily available equipment does not alone increase PA levels in this group of older adults as compared to receiving PA counselling. We recommend that future studies combine resistance training with walking, balance training, and/or behavioral strategies to affect muscle strength, physical function, and PA level in older adults. Furthermore, future studies should continue to investigate the entire intensity spectrum, from SB to MVPA, in older adults.

## Figures and Tables

**Table 1:** Baseline characteristics of the participants.

Characteristics	RTG (*n* = 64)	CG (*n* = 43)	ICC
Age (years), median (IQR)	87 (80–90)	86 (80–90)	
Women, *n* (%)	42 (66)	22 (51)	
Use of walking devices, *n* (%) *	33 (52)	31 (72)	
Height (cm), mean (SD)	160 (9)	164 (9)	
Weight (kg), median (IQR)	66.5 (55.5–79.5) ^a^	70.4 (62.4–80.2) ^b^	
Body Mass Index (kg/m^2^), median (IQR)	25.1 (23.6–28.1) ^a^	27.0 (23.7–30.3) ^b^	
Wear time (min/day), mean (SD) ^§^	805 (77) ^c^	817 (70) ^d^	
Number of valid days, median (IQR) ^§^	13 (12–14) ^c^	13 (11–14) ^d^	
TPA (cpm), mean (SD)	278 (165) ^c^	224 (138) ^d^	0.16
SB (min/day), mean (SD)	600 (100) ^c^	643 (85) ^c^	0.10
LPA (min/day), mean (SD)	170 (73) ^c^	145 (64) ^c^	0.10
MVPA (min/day), mean (SD)	35 (35) ^c^	29 (30) ^d^	0.16
Steps (steps/day), mean (SD)	6623 (3258) ^c^	5223 (2623) ^d^	0.18

RTG, resistance training group; CG, control group; ICC, intra cluster correlation; IQR, interquartile range; SD, standard deviation; TPA, total physical activity; cpm, counts per minute; SB, sedentary behavior; LPA, light physical activity; MVPA, moderate-to-vigorous physical activity. * Includes walker or crutches, one participant in CG with missing data. **^§^** Minutes of wear and valid days of accelerometer wear. ^a^ *n* = 63 ^b^ *n* = 40 ^c^ *n* = 60 ^d^ *n* = 39.

## Data Availability

The datasets used and/or analyzed during the current study are available from the corresponding author on reasonable request.

## Supplementary Materials

The following are available online at https://www.mdpi.com/article/10.3390/ijerph18136682/s1, Supplementary Table S1—Per-protocol analysis of participants with ≥60% attendance to training. Supplementary Table S2—Sensitivity analysis including participants above 70 years. Supplementary Table S3—Sensitivity analysis with adjustment for baseline value of the outcome and without combined baseline. Supplementary Table S4—Sensitivity analysis with adjustment for body mass index, age, and accelerometer wear time.
