# Supplementary material for: Physical Activity Level Following Resistance Training in Community-Dwelling Older Adults Receiving Home Care: Results from a Cluster-Randomized Controlled Trial
Source: Int J Environ Res Public Health. 2021 Jun 22;18(13):6682. doi: 10.3390/ijerph18136682 (PMC8297335; doi:10.3390/ijerph18136682)
Supplement: Supplementary file 1 [file ijerph-18-06682-s001.zip › Table S1.pdf]

Table S1. Per protocol analysis including participants with ≥60% attendance to training.

Outcome	n	Baseline Mean (95% CI)	4 months RTG Mean (95% CI)	4 months CG Mean (95% CI)	Between-group difference Mean (95% CI)	*p*	8 months RTG Mean (95% CI)	8 months CG Mean (95% CI)	Between-group difference Mean (95% CI)	*p*
TPA (cpm)	66	270 (221-319)	249 (196-302)	242 (189-294)	7 (30-44)	0.699	270 (216-324)	240 (187-292)	30 (-9-69)	0.127
SB (min/day)	66	621 (591-651)	630 (593-667)	636 (600-672)	-6 (-43-32)	0.762	616 (578-667)	633 (596-669)	-16 (-56-24)	0.424
LPA (min/day)	66	158 (142-175)	153 (132-175)	147 (127-168)	6 (-17-29)	0.609	165 (142-188)	150 (129-171)	15 (-10-39)	0.242
MVPA (min/day)	66	36 (26-46)	32 (22-43)	32 (21-42)	0 (-6-8)	0.827	35 (24-46)	30 (20-41)	5 (-2-12)	0.191
Steps (steps/day)	66	6108 (5205-7017)	5388 (4363-6419)	5632 (4616-6647)	-244 (-1114-626)	0.583	6120 (5063-7178)	5937 (4916-6959)	183 (-734-1100)	0.696

Estimated means and 95% confidence intervals (95% CI) using linear mixed models (unadjusted model). RTG, resistance training group; CG, control group; TPA, total physical activity; cpm, counts per minute; SB, sedentary behavior; LPA, light physical activity; MVPA, moderate-to-vigorous physical activity.
