# Supplementary material for: Physical Activity Level Following Resistance Training in Community-Dwelling Older Adults Receiving Home Care: Results from a Cluster-Randomized Controlled Trial
Source: Int J Environ Res Public Health. 2021 Jun 22;18(13):6682. doi: 10.3390/ijerph18136682 (PMC8297335; doi:10.3390/ijerph18136682)
Supplement: Supplementary file 1 [file ijerph-18-06682-s001.zip › Table S2.pdf]

Table S2. Sensitivity analysis including participants above 70 years.

Outcome	n	Baseline Mean (95% CI)	4 months		Between-group difference		8 months		Between-group difference	
			RTG Mean (95% CI)	CG Mean (95% CI)	Mean (95% CI)	*p*	RTG Mean (95% CI)	CG Mean (95% CI)	Mean (95% CI)	*p*
TPA (cpm)	94	268 (220-315)	238 (188-288)	239 (187-292)	-1 (-35-32)	0.925	254 (202-306)	237 (184-289)	17 (-20-54)	0.367
SB (min/day)	94	612 (588-639)	623 (596-651)	629 (598-661)	-6 (-37-25)	0.721	614 (583-645)	625 (593-656)	-11 (-45-24)	0.542
LPA (min/day)	94	163 (145-181)	151 (131-171)	153 (130-175)	-2 (-22-19)	0.885	162 (140-184)	156 (133-179)	6 (-16-29)	0.583
MVPA (min/day)	94	35 (25-45)	30 (19-40)	30 (19-41)	0 (-7-6)	0.879	32 (21-43)	29 (18-40)	3 (-4-10)	0.376
Steps (steps/day)	94	6185 (5309-7060)	5354 (4404-6303)	5701 (4687-6715)	-347 (-1131-436)	0.385	5889 (4881-6896)	6016 (4995-7036)	-127 (-988-735)	0.385

Estimated means and 95% confidence intervals (95% CI) using linear mixed models (unadjusted model). RTG, resistance training group; CG, control group; TPA, total physical activity; cpm, counts per minute; SB, sedentary behavior; LPA, light physical activity; MVPA, moderate-to-vigorous physical activity.
