# Supplementary material for: Physical Activity Level Following Resistance Training in Community-Dwelling Older Adults Receiving Home Care: Results from a Cluster-Randomized Controlled Trial
Source: Int J Environ Res Public Health. 2021 Jun 22;18(13):6682. doi: 10.3390/ijerph18136682 (PMC8297335; doi:10.3390/ijerph18136682)
Supplement: Supplementary file 1 [file ijerph-18-06682-s001.zip › Table S3.pdf]

Table S3. Sensitivity analysis without combined baseline, but adjusted for baseline value of the outcome.

Outcome	n	Baseline		4 months		Between-group difference		8 months		Between-group difference		p-value		
		RTG Mean (95% CI)	CG Mean (95% CI)	RTG Mean (95% CI)	CG Mean (95% CI)	Mean (95% CI)	*p*	RTG Mean (95% CI)	CG Mean (95% CI)	Mean (95% CI)	*p*	Group	Time	Group ×Time
TPA (cpm)	99	263 (249-277)	258 (241-275)	239 (233-255)	228 (207-248)	11 (-14-37)	0.390	249 (229-268)	230 (209-250)	19 (-9-48)	0.187	0.180	0.000	0.702
SB (min/day)	99	610 (588-632)	636 (609-662)	620 (596-643)	651 (621-681)	-31 (-69-7)	0.109	614 (587-641)	642 (612-672)	-28 (-68-13)	0.180	0.087	0.287	0.947
LPA (min/day)	99	163 (155-171)	159 (149-169)	149 (140-159)	151 (139-163)	-2 (-17-13)	0.799	162 (150-174)	151 (139-164)	11 (-7-28)	0.229	0.401	0.075	0.511
MVPA (min/day)	99	34 (31-37)	33 (30-37)	30 (27-38)	28 (24-32)	2 (-3-7)	0.498	31 (27-35)	28 (24-32)	3 (-2-9)	0.245	0.290	0.001	0.692
Steps (steps/day)	99	6204 (5904-6503)	6052 (5677-6426)	5440 (5029-5787)	5642 (5189-6096)	-202 (-777-372)	0.490	5904 (5463-6345)	5860 (5399-6321)	44 (-599-686)	0.894	0.990	0.003	0.589

Estimated means and 95% confidence intervals (95% CI) using linear mixed models (adjusted model 1). RTG, resistance training group; CG, control group; TPA, total physical activity; cpm, counts per minute; SB, sedentary behavior; LPA, light physical activity; MVPA, moderate-to-vigorous physical activity.
