# Supplementary material for: Physical Activity Level Following Resistance Training in Community-Dwelling Older Adults Receiving Home Care: Results from a Cluster-Randomized Controlled Trial
Source: Int J Environ Res Public Health. 2021 Jun 22;18(13):6682. doi: 10.3390/ijerph18136682 (PMC8297335; doi:10.3390/ijerph18136682)
Supplement: Supplementary file 1 [file ijerph-18-06682-s001.zip › Table S4.pdf]

Table S4. Sensitivity analysis with adjustment for body mass index, age, and accelerometer wear time.

Outcome	n	Baseline Mean (95% CI)	4 months		Between-group difference		8 months		Between-group difference	
			RTG Mean (95% CI)	CG Mean (95% CI)	Mean (95% CI)	*p*	RTG Mean (95% CI)	CG Mean (95% CI)	Mean (95% CI)	*p*
TPA (cpm)	100	264 (218-309)	241 (193-289)	236 (185-286)	5 (-28-38)	0.757	257 (206-307)	238 (187-290)	18 (-19-56)	0.343
SB (min/day)	100	616 (591-641)	630 (603-657)	629 (601-658)	1 (-21-22)	0.958	617 (588-645)	628 (599-657)	-11 (-36-13)	0.368
LPA (min/day)	100	161 (145-178)	151 (133-170)	152 (131-172)	-1 (-19-18)	0.953	163 (143-184)	153 (131-174)	10 (-10-31)	0.325
MVPA (min/day)	100	34 (25-43)	30 (21-4 0)	30 (20-40)	0 (-6-7)	0.530	32 (22-42)	31 (21-41)	1 (-6-8)	0.728
Steps (steps/day)	100	6146 (5350-6943)	5477 (4623-6332)	5700 (4773-6626)	-222 (-929-484)	0.538	5990 (5067-6912)	6001 (5058-6946)	-12 (-825-801)	0.977

Estimated means and 95% confidence intervals (95% CI) using linear mixed models (adjusted model 2). RTG, resistance training group; CG, control group; TPA, total physical activity; cpm, counts per minute; SB, sedentary behavior; LPA, light physical activity; MVPA, moderate-to-vigorous physical activity.
